# Evolution of woodcutting behaviour in Early Pliocene beaver driven by consumption of woody plants

**DOI:** 10.1038/s41598-020-70164-1

**Published:** 2020-08-04

**Authors:** Tessa Plint, Fred J. Longstaffe, Ashley Ballantyne, Alice Telka, Natalia Rybczynski

**Affiliations:** 1 Department of Earth Sciences, The University of Western Ontario, London, ON N6A 5B7 Canada; 2 Present Address: The Lyell Centre, Heriot-Watt University, Edinburgh, EH14 4AS UK; 3 Department of Ecosystem and Conservation Sciences, University of Montana, Missoula, MT 59812 USA; 4 Paleotec Services, 574 Somerset St W Suite 1, Ottawa, ON K1R 5K2 Canada; 5 Department of Palaeobiology, Canadian Museum of Nature, Ottawa, ON K1P 6P4 Canada; 6 Department of Biology, Department of Earth Sciences, Carleton University, Ottawa, ON K1S 5B6 Canada

**Keywords:** Palaeoecology, Biogeochemistry, Phylogenetics

## Abstract

Modern beavers (*Castor*) are prolific ecosystem engineers and dramatically alter the landscape through tree harvesting and dam building. Little is known, however, about the evolutionary drivers of their woodcutting behaviour. Here we investigate if early woodcutting behaviour in Castoridae was driven by nutritional needs. We measured stable carbon and nitrogen isotopes (*δ*^13^C and *δ*^15^N) of coeval subfossil plants and beaver collagen (*Dipoides* sp.) from the Early Pliocene, High Arctic Beaver Pond fossil locality (Ellesmere Island), in order to reconstruct *Dipoides* sp. diet. Isotopic evidence indicates a diet of woody plants and freshwater macrophytes, supporting the hypothesis that this extinct semiaquatic beaver engaged in woodcutting behaviour for feeding purposes. In a phylogenetic context, the isotopic evidence implies that woodcutting and consumption of woody plants can be traced back to a small-bodied, semiaquatic Miocene castorid, suggesting that beavers have been consuming woody plants for over 20 million years. We propose that the behavioural complex (swimming, woodcutting, and consuming woody plants) preceded and facilitated the evolution of dam building. Dam building and food caching behaviours appear to be specializations for cold winter survival and may have evolved in response to late Neogene northern cooling.

## Introduction

Beavers today are renowned for their woodcutting behaviour and construction abilities. The extant genus *Castor* harvests trees and shrubs for sustenance (particularly during the winter^1^), but also for the purpose of lodge and dam building. These behaviours have a profound effect on regional hydrology, nutrient flow across the landscape, and local biodiversity, thus making them exemplary “ecosystem engineers”^2–4^. Modern beavers also are known to feed on trees. This food source is particularly important for northern populations that survive freezing winters, subsisting on their underwater caches of leafy branches^5,6^.

Castoridae is a diverse family of herbivorous Holarctic rodents, originating during the late Eocene^7–9^. The only definitive evidence of woodcutting in extinct beavers is from the Pliocene-aged High Arctic Beaver Pond fossil site, which preserves evidence of beaver-cut wood and a possible dam-core associated with the extinct beaver genus, *Dipoides* (species not yet described)^9–11^. Examination of *Dipoides* sp. incisors and cut marks on wood from the Beaver Pond site suggest that *Dipoides* sp. woodcutting performance was poorer than that of modern *Castor*^11^. The appearance of woodcutting in *Dipoides* sp., a distant relative of *Castor*, implies that this behaviour originated 20 to 24 Ma ago, in a group of semiaquatic beavers that includes both extant species (C*astor canadensis* and *Castor fiber*), the small Holarctic genus *Dipoides*, and the North American Ice Age giant beaver *Castoroides*^7,9,12^. And yet recent research shows that the diet of Ice Age *Castoroides*, a close relative of *Dipoides*, was dominated by submerged plants, not trees and shrubs^13^. This finding, alongside a lack of definitive evidence of lodges or dams at the Beaver Pond site highlights the question: Does the cut wood at the Beaver Pond site represent a means of gathering food?

**Figure 1:**
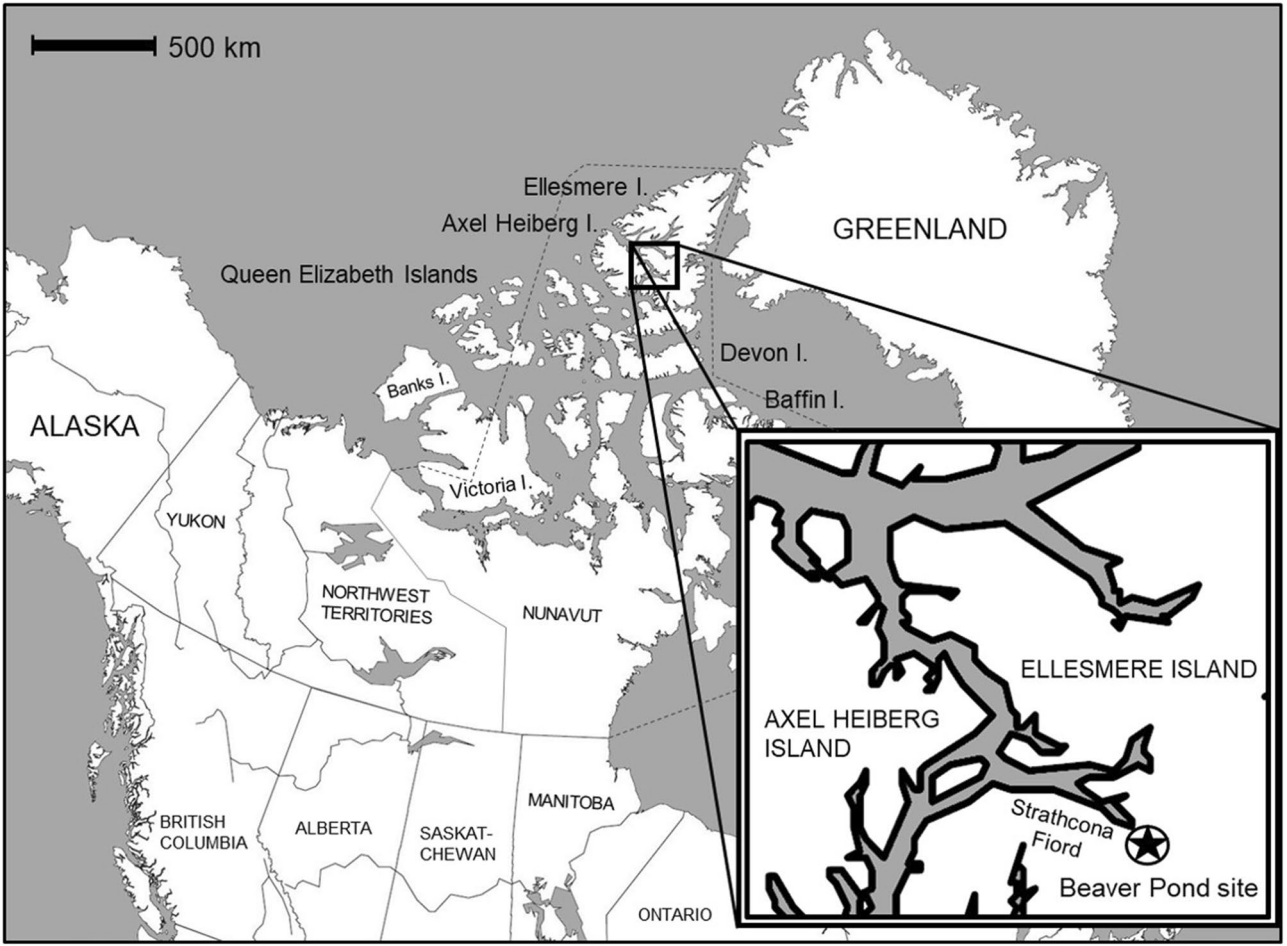
Map of the Canadian Arctic Archipelago. The Beaver Pond site is located near the head of Strathcona Fiord, Ellesmere Island (78° 33′ N, 82° 25′ W). Base map created in Adobe Illustrator (version 24.2.1), https://www.adobe.com/uk/products/illustrator.html.

Here, we present stable carbon and nitrogen isotope data for Pliocene-age (i) plant macrofossils and (ii) bone collagen from High Arctic *Dipoides* sp. subfossil remains. The specimens originate from a peat deposit at the Beaver Pond fossil site, located on Ellesmere Island (locally known as Umingmak Nuna, meaning “land of muskoxen”), situated within the Canadian Arctic Archipelago (78° 33′ N, 82° 25′ W) (Fig. 1). We reconstruct High Arctic *Dipoides* sp. palaeodiet within the context of coeval terrestrial and freshwater plant macrofossil remains excavated from the same ~ 4 Ma old peat layer. The Beaver Pond site provides a very rare opportunity for such a palaeodiet reconstruction using coeval herbivore and plant remains.

### The Beaver Pond fossil site

**Figure 2:**
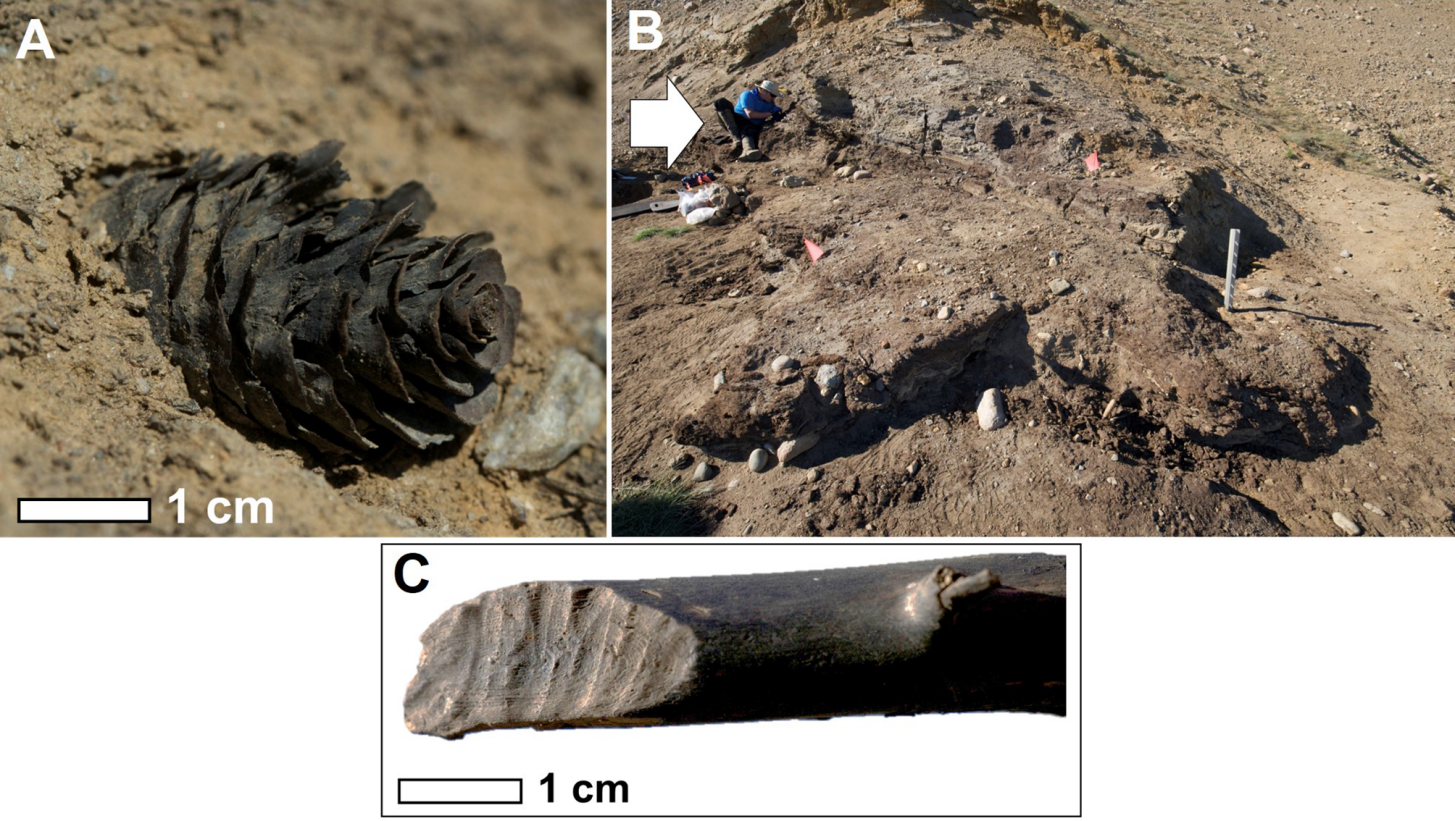
(**A**) An in-situ macrofossil cone within the Beaver Pond fossiliferous peat deposit. Scale bar is 1 cm. (**B**) Excavation of the peat deposit at the Beaver Pond site (2008), Strathcona Fiord, Ellesmere Island. White arrow indicates person for scale. (**C**) A beaver-cut stick excavated from the Beaver Pond site. Cut marks produced by *Dipoides* sp. Photographs by M. Lipman.

The Beaver Pond site is a succession of fossiliferous peat deposits interspersed within 40 m of sandy, cross-bedded, fluvial deposits and capped with glacial till^14^. The thickest peat deposit within this sequence, which yielded material for this study, sits 380 m above present day sea-level overlooking Strathcona Fiord. Most recent terrestrial cosmogenic nuclide burial dating of sands above this peat layer have yielded a date of 3.9 + 1.5/− 0.5 Ma^15^, placing the minimum age of peat formation during the late Early Pliocene (late Zanclean). This peat unit is characterized by an abundance of beaver-cut sticks^10,11^ and has yielded a wide array of beautifully preserved plant macrofossils, invertebrates, and vertebrate fossil remains (Fig. 2). Rybczynski and Harington^16^, Matthews and Fyles^17^, Hutchison and Harington^18^, Tedford and Harington^10^, Dawson and Harington^19^, Murray et al.^20^, Mitchell et al.^21^, Gosse et al.^22^, and Wang et al.^23^ provide detailed descriptions of Pliocene terrestrial flora and faunal assemblages associated with the Beaver Pond site.

Ellesmere Island today is a polar desert, with sparse flora and very little precipitation^24^. The landscape was very different during the Early Pliocene warm period, when the climate supported wetland habitat surrounded by open larch forest^10,21,22,25^. During the Pliocene, Ellesmere Island was on the eastern edge of a large coastal plain, where intense Neogene thawing and weathering liberated sediment to create a thick, continuous clastic wedge across the Canadian Arctic Archipelago (referred to as the Beaufort Formation in the western Canadian Arctic islands). It is hypothesized that northwest passages did not exist during the Pliocene, as they had yet to be incised by fluvial and glacial erosion^22,26^. This unbroken coastal plain altered ocean circulation patterns in the High Arctic, and along with the Bering Isthmus that connected North American and Eurasia until 5.5 Ma ago^27^ would have enabled the migration of terrestrial species across the Neogene High Arctic^10^.

Late Early Pliocene mean global temperature was 2–3 °C above modern^28^ and high latitude regions experienced amplified warming. Pliocene Arctic mean annual temperature was near freezing, which is ~ 15–22 °C warmer than present day, and tree line was ~ 2000 km further north^15,24,29–33^. Summer temperatures at the Beaver Pond site reached 20 °C, while winter temperatures were more moderate than present day, with a low of ~ – 12 °C^24^. Despite the relatively mild conditions, the Beaver Pond site still experienced total darkness and subzero temperatures during the winter months.

There is no modern analog for the ecological community found at the Beaver Pond site, although the flora of present-day Labrador (Canada) is considered to be similar^22,34–36^. The Beaver Pond macrofossil assemblage indicates a larch forest, although birch, alder, spruce, pine, cedar, and cold-adapted woody shrubs were also present (see Matthews and Fyles^17^ for a complete list of identified flora). The Beaver Pond site supported higher faunal biodiversity than any modern near-tree line communities.

The remains of a complex faunal community were discovered at the Beaver Pond site, including beaver (*Dipoides* sp., see below), three-toed horse, bear, badger, “deerlet”, water fowl, fish and a rabbit relative^10,22^. Pliocene-age sites with similar fauna and flora community composition are very rare—Idaho in mid-continent North America, and the high altitude Yushe Basin, in northeastern China are the only known sites with similar (but not equivalent) faunal assemblages^37,38^.

### Dipoides ecology

The most common vertebrate remains at the Beaver Pond site belong to *Dipoides*, an extinct genus of beaver known from the Neogene of Eurasia and North America, represented by 12 different species^7,39–41^. Although not directly related to the extant *Castor*, both genera share semiaquatic and woodcutting behaviours^9,11,42^.

The Beaver Pond site is the only known locality with sufficiently well-preserved plant macrofossils to record evidence of *Dipoides* sp. woodcutting behaviour. The peat deposits are hypothesized to be the remnants of an ancient beaver pond due to the presence of many beaver-cut sticks, and even a cluster of cut sticks, cobbles, and silt that resemble the core of a beaver dam^10^.

Here we use stable isotope data to understand *Dipoides* sp. diet and elucidate the purpose of their woodcutting behaviour. Our study aims to describe the relative contributions of woody vegetation and aquatic plants to the diet of *Dipoides* sp. using stable carbon and nitrogen isotope analysis of contemporary sub-fossil skeletal and plant macrofossil material from the Beaver Pond site. This new information is used to better interpret (i) the ecological impact of *Dipoides* sp. on the Pliocene landscape, (ii) *Dipoides* sp. potential for winter survival strategies such as underwater food caching, and (iii) the evolutionary context of tree-exploitation within the Castoridae family.

### Stable isotopes and palaeodiet

The stable carbon (*δ*^13^C) and nitrogen (*δ*^15^N) isotope compositions of an animal’s bodily tissues correlate closely with that of its diet, when adjusted for ^13^C- and ^15^N-enrichment that occurs during collagen formation and with each successive trophic level^43,44^. Well-preserved bone collagen is therefore a useful integrator of an animal’s diet. In addition, sufficient context is required to accurately describe the nutrient flow between subsequent trophic levels of an ecosystem. In particular, the diet of an organism must be interpreted within the context of an appropriate dietary baseline. This baseline is composed of isotopically defined food or “menu-items” available to the organism.

The isotopic composition of primary producers at the base of the food chain control the *δ*^13^C and *δ*^15^N of the dietary baseline for herbivores. The *δ*^13^C and *δ*^15^N of primary producers depend on physiology (i.e. which photosynthetic pathway the plant employs) and the isotopic composition of bioavailable sources of C and N (i.e. atmospheric CO_2_). Casey and Post^45^ provide a thorough review of how primary producer *δ*^13^C and *δ*^15^N vary with physiology and local terrestrial and aquatic environmental conditions. A particular challenge in many forested-wetland environments, however, is that the carbon and nitrogen isotope range of terrestrial and freshwater plants overlap. There are, however, sufficient differences between the *δ*^13^C and *δ*^15^N of terrestrial vegetation utilizing the C3-photosynthetic pathway and vascular freshwater plants (macrophytes) for them to serve as useful endmembers of herbivore diet in such environments (see Methodology).

Another challenge is that the isotopic composition of regional and global C and N baselines (and subsequently, that of primary producers that use them) can change over time^46,47^. Hence, reconstructing the diet of herbivores that lived thousands or millions of years ago can be problematic when using isotopic data, as is very rare to find sufficiently preserved coeval plant material and faunal remains from the same geologic locality. Typically, isotopic data for modern plants are all that are available in palaeodiet studies. Fortunately, much of this concern is alleviated at the Beaver Pond site, given the excellent organic preservation of coeval plant and animal tissues.

## Results

### Bone collagen stable carbon and nitrogen isotopes

**Figure 3:**
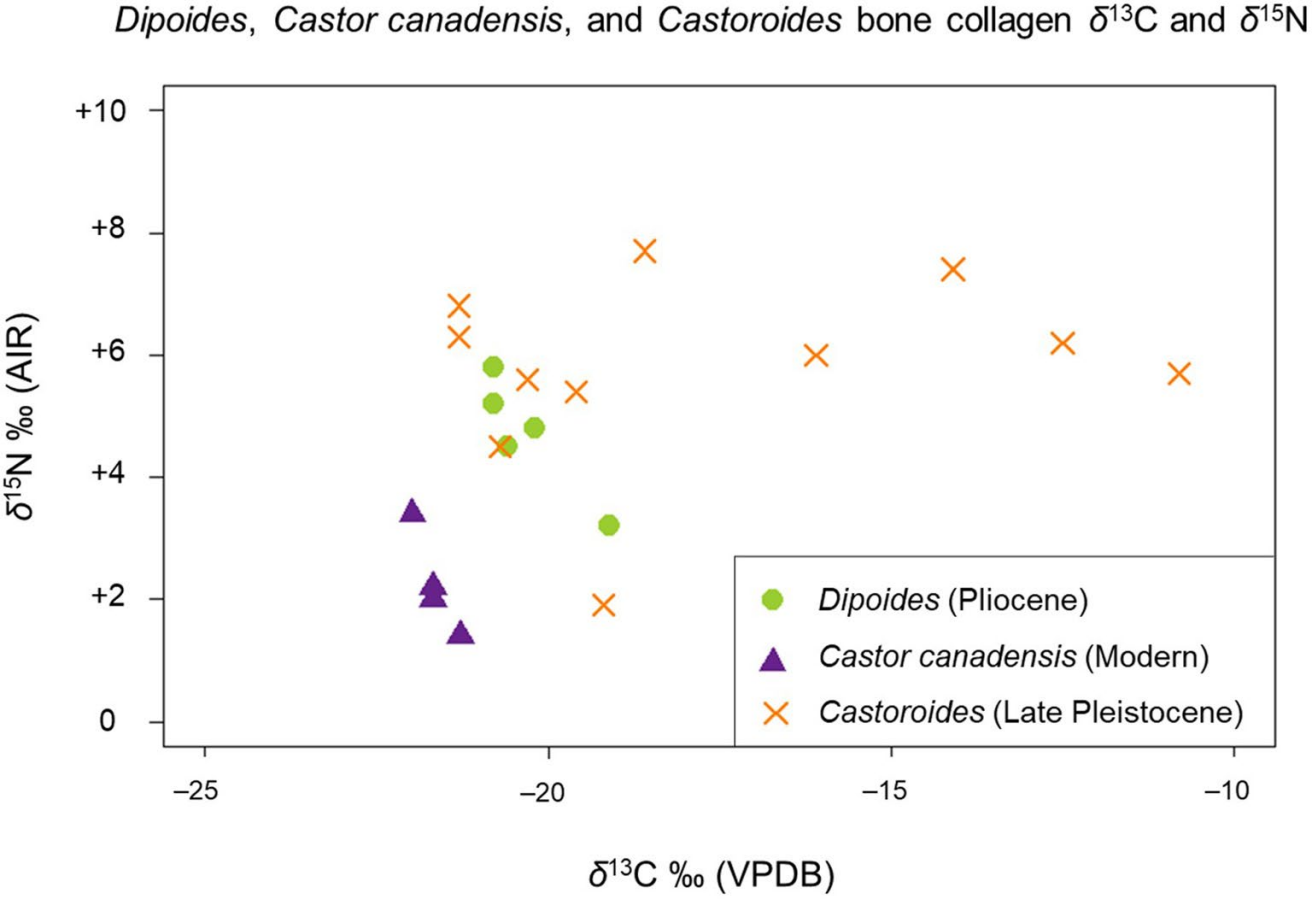
Comparison of bone collagen *δ*^13^C and *δ*^15^N among Pliocene *Dipoides* sp. (4 Ma, from the Beaver Pond Site, Ellesmere Island), modern *Castor canadensis* (collected 2013–2014, from Yukon Territory), and Pleistocene *Castoroides* (late Pleistocene, from Beringia, Yukon Territory and Ohio, USA). *Castor canadensis* and *Castoroides* carbon isotope compositions are corrected for Suess effects appropriate to their time period (see text). *Castor canadensis* and *Castoroides* isotope data from Plint et al.^13^.

*Dipoides* sp. (n = 5) bone collagen stable isotope results (*δ*^13^C_col_ and *δ*^15^N_col_) are presented in Table 1. *Dipoides* sp. *δ*^13^C_col_ ranges from − 20.8 to − 19.1‰, with a mean of − 20.3‰, and *δ*^15^N_col_ ranges from + 3.2 to + 5.8‰, with a mean of + 4.7‰ (Fig. 3).

**Table 1:** Beaver Pond site *Dipoides* sp.*, modern *Castor canadensis*, and Pleistocene *Castoroides* bone collagen *δ*^13^C, *δ*^15^N, and preservation parameter data.

Project sample ID	*δ*^13^C ‰ (VPDB)	*δ*^15^N ‰ (AIR)	C (wt%)	N (wt%)	Collagen yield (%)	Atomic C:N ratio	Taxon	Skeletal element
NuFV 292	− 19.1	+ 3.2	39.3	13.5	No data	3.4	*Dipoides* sp.	Humerus
NuFV 305	− 20.8	+ 5.8	40.4	14.7	No data	3.2	*Dipoides* sp.	Tibia fragment
CMN 51766	− 20.2	+ 4.8	39.7	13.2	No data	3.5	*Dipoides* sp.	Tibia fragment
CMN 51768	− 20.6	+ 4.5	41.8	14.7	No data	3.3	*Dipoides* sp.	Tibia and fibula
CMN 51769	− 20.8	+ 5.2	39.3	13.2	No data	3.5	*Dipoides* sp.	Tibia and fibula
B2-TP2013-A	− 24.0	+ 3.4	44.8	16.9	10.2	3.1	*Castor canadensis*	Metapodial
B6-TP2014-1	− 23.7	+ 2.0	43.7	16.8	16.7	3.0	*Castor canadensis*	Mandible
B7-TP2014	− 23.3	+ 1.4	43.1	16.6	16.6	3.0	*Castor canadensis*	Mandible
B8-TP2014-1	− 23.7	+ 2.2	42.3	16.2	16.3	3.0	*Castor canadensis*	Mandible
CMN 16657	− 21.2	+ 6.3	41.7	15.6	1.4	3.1	*Castoroides*	Humerus
CMN 18306	− 19.1	+ 1.9	41.5	15.2	1.4	3.2	*Castoroides*	Pelvis
CMN 18707	− 10.7	+ 5.7	41.8	15.5	3.6	3.1	*Castoroides*	Tibia
CMN no ID	− 18.5	+ 7.7	41.2	15.7	No data	3.1	*Castoroides*	Femur
CMN 14711	− 16.0	+ 6.0	33.1	11.9	1.1	3.2	*Castoroides*	Humerus
CMN 14781	− 14.0	+ 7.4	39.6	14.5	No data	3.2	*Castoroides*	Long bone diaphysis
CMN 33640	− 12.4	+ 6.2	39.7	14.4	1.4	3.2	*Castoroides*	Humerus
CMN 43178	− 21.2	+ 6.8	42.7	15.5	1.4	3.2	*Castoroides*	Femur
OHS N9109	− 20.2	+ 5.6	35.5	12.6	3.6	3.3	*Castoroides*	Mandible
OHS N9087	− 20.6	+ 4.5	40.0	14.3	No data	3.3	*Castoroides*	Mandible
OHS N8739	− 19.5	+ 5.4	37.4	13.5	1.1	3.2	*Castoroides*	Incisor (dentin)

*C. canadensis* specimens collected in 2013–2014 from Yukon Territory, Canada. *Castoroides* specimens collected from localities in Yukon Territory (Beringia), Canada, and Ohio, USA. Values in bold indicate the mean value where duplicate analyses were completed for the same specimen. No Suess effect correction applied to the reported stable carbon isotope compositions. *Castor canadensis* and *Castoroides* data from Plint et al.^13^^.^

*Dipoides sp. data courtesy of Paul Matheus.

Atomic C:N ratio and carbon and nitrogen contents (wt%) were used to assess collagen preservation for *Dipoides* skeletal material (Table 1). All specimen parameters are within the accepted range for well-preserved archaeological or palaeontological skeletal material from temperate or polar regions reported by van Klinken^48^ (acceptable parameters: C wt% = 34.8 ± 8.8%; N wt% =  ~ 11–16%; atomic C:N = 3.1–3.5).

### Plant macrofossil species diversity

By volume, the Beaver Pond peat sample examined in this study consisted of 85% bryophytes, 10% wood and twigs, and 5% macrofossils. Eleven genera were identified, representing a diverse assemblage of terrestrial and freshwater plants (Table 2). Seven taxa were analyzed for stable carbon and nitrogen isotope compositions (*Scorpidium scorpioides*, *Larix*, *Betula, Stuckenia filiformis*, *Scheuchzeria* sp., *Cornus sericea*, *Menyanthes trifoliata*) (Table 2). The dominant moss type was *Scorpidium scorpioides* (hooked scorpion moss). *Larix* (larch—a deciduous conifer) was the only conifer species identified from this peat sample (although many other tree species have been previously identified from the Beaver Pond site—see Introduction). Plant macrofossils from multiple genera (*Myrica*, *Shepherdia*, *Potamogeton*, and *Hippuris*) and from three species of *Carex* (*Carex aquatilis*, *Carex diandra*, and *Carex maritime*) were also recognized, but in insufficient quantities for stable isotope analysis.

**Table 2:** Beaver Pond site plant macrofossil taxonomic identification, *δ*^13^C, *δ*^15^N, and C and N (wt%) abundances.

Project sample ID	*δ*^13^C ‰ (VPDB)	*δ*^15^N ‰ (AIR)	C (wt%)	N (wt%)	C/N (wt%)	Atomic C:N	Taxon	Common name	Description and sample treatment
1	− 36.6	+ 4.8	36.8	0.5	77.4	90.25	*Scorpidium scorpioides*	Hooked scorpion moss	Bulk plant, dry pick cleaning, no ultrasonic water bath
2	− 35.3	+ 4.7	41.9	0.6	76.9	89.64	*Scorpidium scorpioides*	Hooked scorpion moss	Bulk plant
2 MET DUP	− 34.6	+ 4.8	41.5	0.6	69.1	80.5	*Scorpidium scorpioides*	Hooked scorpion moss	Bulk plant
3	− 25.1	+ 3.7	45.5	0.9	57.7	67.3	*Larix*	Larch	Scales from single dissected cone
5	− 25.4	+ 3.8	45.0	0.8	57.5	67.1	*Larix*	Larch	Short shoots
7	− 23.3	+ 2.1	48.0	0.3	140.7	164.0	*Larix*	Larch	Seeds from single dissected cone
8	− 23.6	+ 2.3	46.9	0.3	145.7	169.9	*Larix*	Larch	Seeds (whole)
9	− 22.7	+ 1.8	47.7	0.3	155.8	181.7	*Larix*	Larch	Seeds (split)
10	− 26.8	+ 2.7	47.4	0.9	54.0	63.0	*Betula*	Arboreal birch	Birch cone bracts
11	− 26.7	Insufficient material	42.0	0.9	47.4	55.3	*Betula*	Dwarf birch	Birch cone bracts
14	− 26.5	+ 2.5	49.3	0.4	122.4	142.7	*Stuckenia filiformis*	Pondweed	Seeds
16	− 26.0	+ 0.2	45.4	1.5	29.5	34.4	*Scheuchzeria* sp.	Pod grass	Seeds
17	− 28.8	+ 0.1	50.9	0.7	72.6	84.7	*Cornus sericea*	Red osier dogwood	Seeds
18	− 24.4	+ 2.4	49.5	0.6	88.6	103.3	*Menyanthes trifoliata*	Bogbean	Seeds
18 MET DUP	− 24.1	+ 2.7	49.3	0.6	88.8	103.6	*Menyanthes trifoliata*	Bogbean	Seeds
19	− 27.1	+ 2.8	48.5	1.0	49.7	58.0	*Betula*	Birch	Twig with bark
21	− 29.2	+ 4.0	13.8	0.6	24.5	28.6	Composed predominantly of moss	Peat	Bulk sample of peat material

Values shown in bold are the average of analytical duplicates. “MET DUP” indicates a method duplicate (see text). All plant macrofossils were cleaned in an ultrasonic water bath, unless otherwise indicated.

### Plant macrofossil stable carbon and nitrogen isotopes

**Figure 4:**
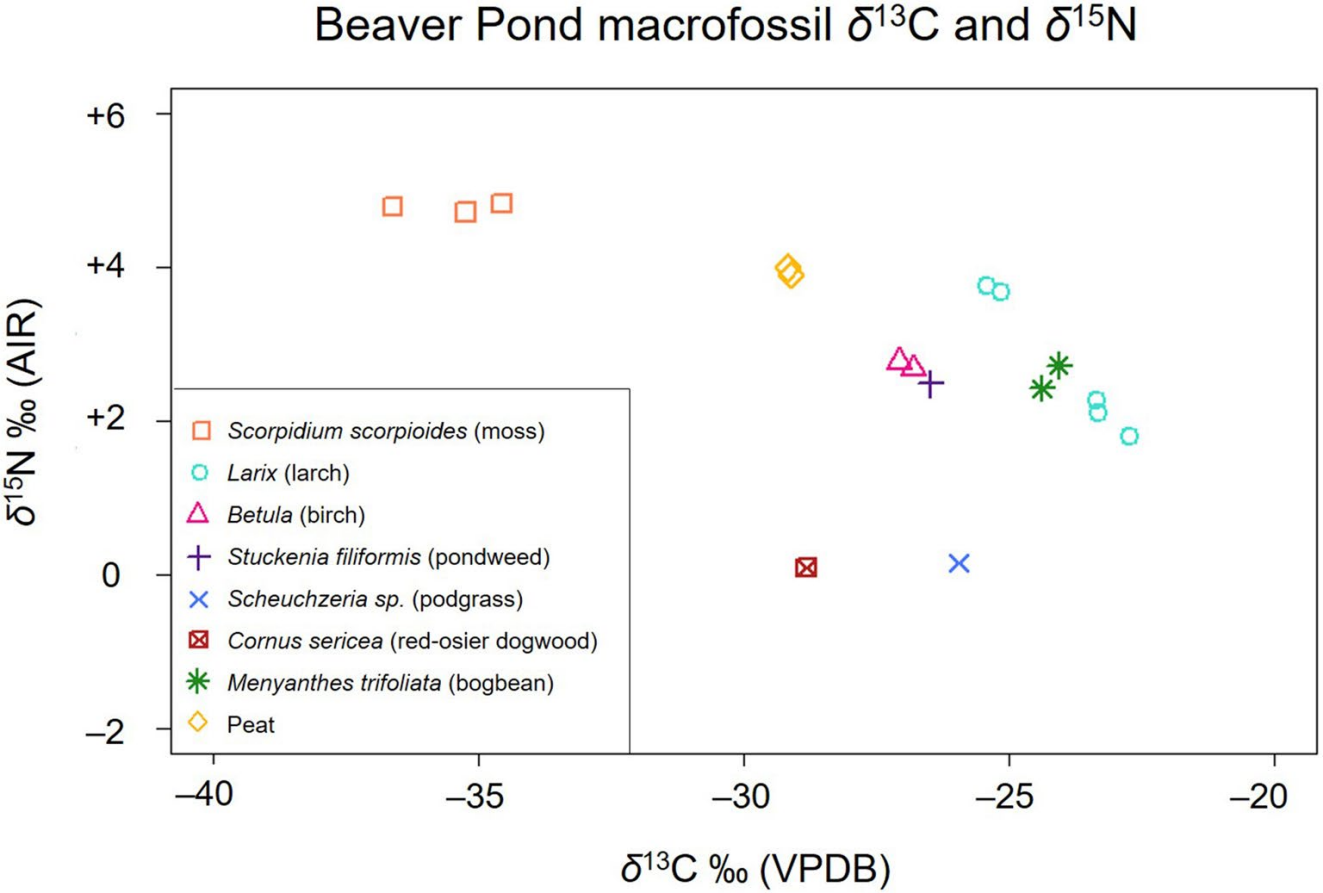
Stable carbon and nitrogen isotope results for the Beaver Pond site plant macrofossils.

**Figure 5:**
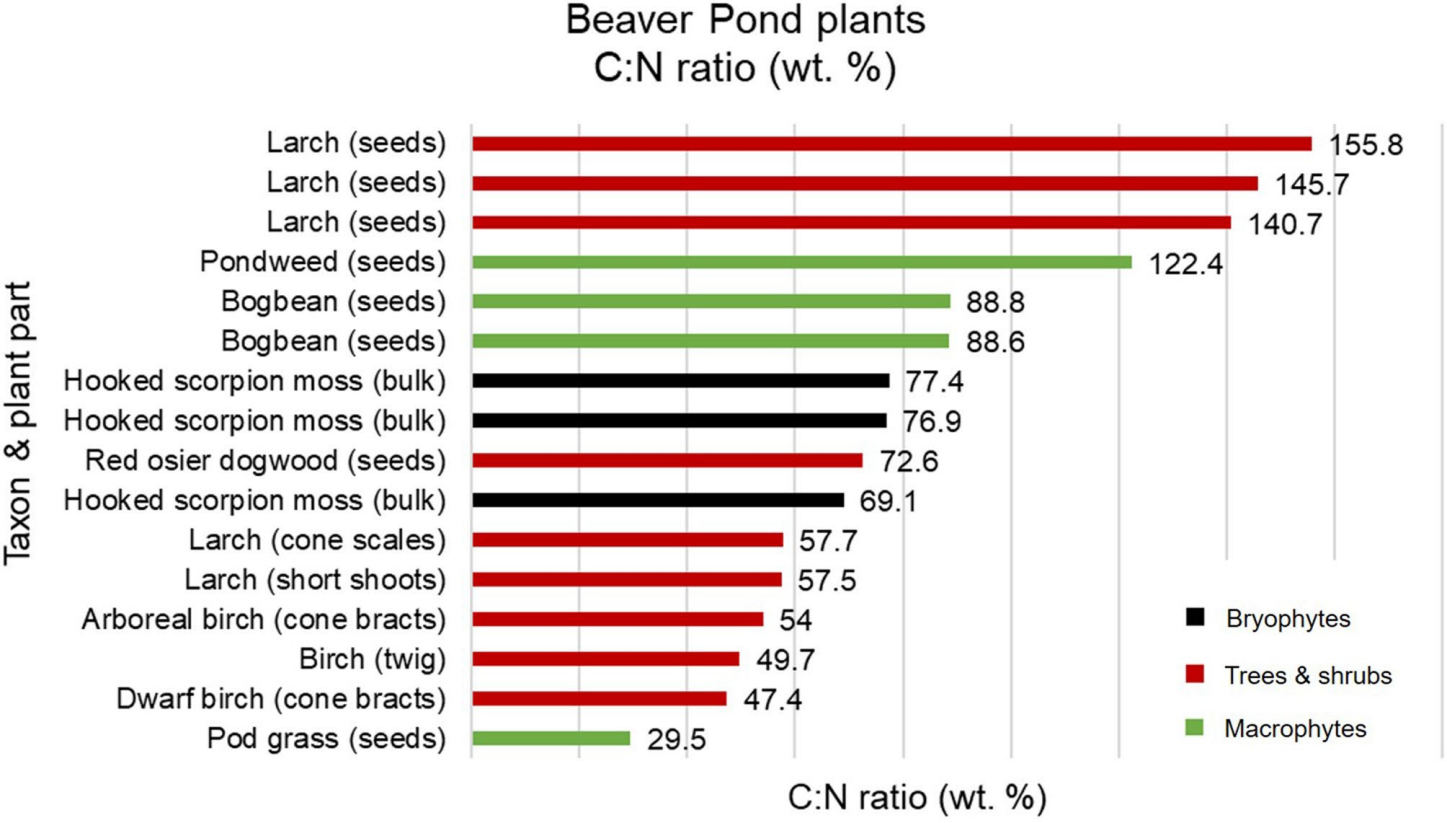
Beaver Pond plant macrofossil carbon and nitrogen content represented by C/N (wt%).

Plant macrofossil stable isotope results are presented in Table 2 and Fig. 4. Macrofossil *δ*^13^C, *δ*^15^N, C (wt%), N (wt%), C/N (wt%), and atomic C:N ratios are all within the range expected for terrestrial and freshwater plants (Table 2, Figs. 4 and 5). Beaver Pond plant macrofossil *δ*^13^C and *δ*^15^N range from − 36.6 to − 22.7‰, and + 0.1 to + 4.8‰, respectively.

The chemical and elemental compositions of plants vary widely by species, life history stage (i.e. senescence), and environment conditions. For these reasons, atomic C:N ratio and carbon and nitrogen content are not considered to be infallible indicators of organic preservation in subfossil plants^49^. That said, the plant macrofossil C (wt%) values obtained in the present study are within or close to the mean carbon content for modern plants (between ~ 40 and 47%) (Metcalfe and Mead^49^, and references therein). Plant macrofossil N (wt%) values are lower than the mean nitrogen contents of modern plants (between ~ 1 and 3%) but are not outside the reported range for modern plants.

### Stable isotope analysis in R (SIAR) mixing model

**Figure 6:**
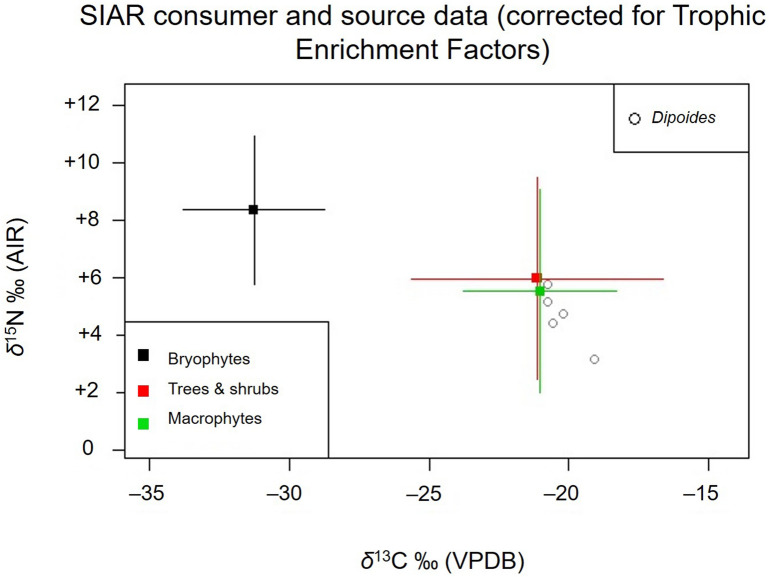
Stable carbon and nitrogen isotope compositions of plant functional groups and *Dipoides* sp. bone collagen generated using SIAR mixing model. Plant functional groupings include terrestrial woody plants (larch, birch, and red-osier dogwood), vascular freshwater macrophytes (pondweed, podgrass, and bogbean), and bryophytes (Hooked scorpion moss). *Dipoides* sp. bone collagen *δ*^13^C and *δ*^15^N are corrected for trophic enrichment factors to render them comparable to the three plant functional groups (represented by their mean and a range of 2SD). The *Dipoides* sp. data, once so corrected, overlap with the plant functional groups that contributed significantly to their diet.

Next, we evaluate *Dipoides* sp. *δ*^13^C_col_ and *δ*^15^N_col_ within the context of the isotopic dietary baseline composed of coeval terrestrial and freshwater vegetation from the High Arctic Beaver Pond fossil site. The faunal and plant macrofossil isotope data were incorporated into a Bayesian mixing model to determine the relative input of terrestrial versus freshwater plants to *Dipoides* sp. diet (Fig. 6). This also allowed us to better assess the connection between *Dipoides* sp. woodcutting behaviour and its consumption of woody plants.

The SIAR model is a statistical tool that uses biotracers (stable isotopes) to estimate the relative input of different sources to a product or mixture. In (palaeo)ecology, mixing models use the stable isotope compositions of different food sources to infer their relative contributions to the composition of overall diet, and assess the probability that the inferred proportions are correct. There are systematic differences in the isotopic composition between a consumer’s collagen and its diet, both for carbon and for nitrogen. Hence, a correction factor must be applied to render data for consumers and possible diet items directly comparable.

Plant macrofossils were divided into three sources, or functional types: terrestrial woody plants, vascular freshwater macrophytes, and bryophytes (mosses). These groupings were created to assess how terrestrial and freshwater resources contributed separately to *Dipoides* sp. diet. The functional types were statistically defined and their relative contribution to diet was assessed using scripts from SIAR V4 in R Studio 3.1.2 (Stable Isotope Analysis in R: An Ecologist’s Guide). Based on existing literature, respective bone collagen-to-diet offsets of + 4.2‰ and + 3.0‰ were subtracted from *Dipoides* sp. *δ*^13^C_col_ and *δ*^15^N_col_ when incorporated into the mixing model^50–53^.

## Discussion

### *Dipoides* sp. palaeoecology

**Figure 7:**
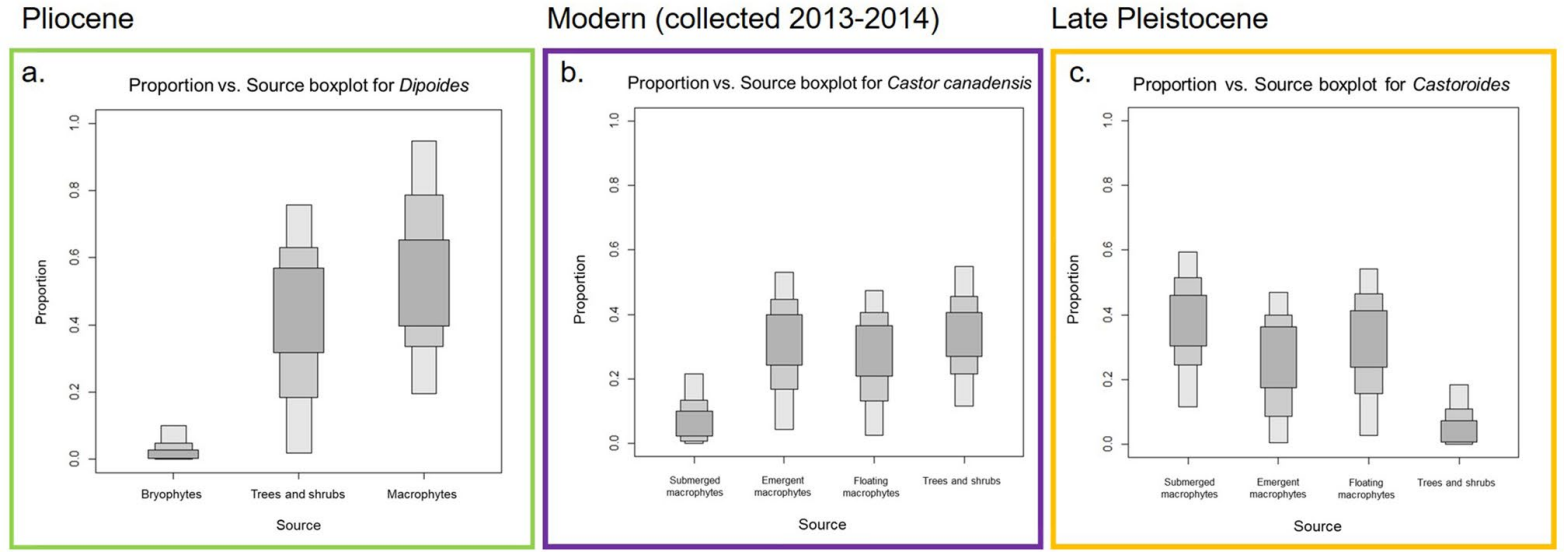
(**a**) Proportion versus Source Boxplot generated using SIAR, indicating the relative proportion that moss, woody vegetation, and aquatic macrophytes contributed to the diet of *Dipoides* sp. at the Beaver Pond site. Darker shaded areas indicate highest probability of source proportion. The Proportion versus Source Boxplots for (**b**) extant *Castor canadensis* and (**c**) late Pleistocene *Castoroides* have been included for comparison. Note the differences in dietary Source data used to distinguish *C. canadensis* and *Castoroides* diet (primarily the sub-division of aquatic plants into categories based on habitat within the water column). b and c from Plint et al.^13^.

The Bayesian mixing model indicates that *Dipoides* sp. consumed both woody plants and freshwater macrophytes in approximately equal proportions (Figs. 6 and 7), although it relied slightly more on freshwater macrophytes. This suggests that *Dipoides* sp. spent a greater proportion of time feeding in the water than on land.

The distribution of *Dipoides* sp. *δ*^13^C_col_ and *δ*^15^N_col_ is not entirely enclosed within the three primary producer functional groups analyzed (Fig. 6). This is likely the result of the relatively small plant macrofossil sample size. Submerged aquatic macrophytes, for example, are under-represented in the plant macrofossils available for stable isotope analysis. Macrophytes have highly variable *δ*^13^C and may have contributed more to *Dipoides* sp. diet than the mixing model suggests. Submerged macrophytes can be highly enriched in ^13^C because of physiological differences (primarily the use of ^13^C-enriched dissolved bicarbonate) or environmental conditions in the water column (i.e. boundary-layer effect)^54–56^. In addition, tree bark is more enriched in ^13^C than tree foliage^57^ and may have been a key resource for *Dipoides* sp.

The results of the dietary mixing model support the interpretation that woody plants were an important contributor to *Dipoides* sp. diet. It is likely that *Dipoides* sp. also used shrubs and trees as a source of construction material^10,11^, but more evidence is needed to confirm this. Similar to extant *Castor*, *Dipoides* sp. may have also demonstrated regional differences in diet, where northern and southern populations utilized different resources according to their availability.

### Nitrogen content and C/N as indicators of forage quality

Plant macrofossil nitrogen content (N wt%) and C/N are indicators of forage quality and may be used to interpret the relative nutrition of dietary inputs. Plants with high N (wt%) contain more protein and energy—likewise, low N (wt%) correlates with low plant digestibility, high fiber and high lignin compound content^58^. Beaver Pond plant macrofossil N (wt%) and C/N are highly variable (Table 2, Fig. 5). Although there is considerable variability in C/N ratios depending upon which plant part was analyzed (i.e. seeds versus woody tissue), woody vegetation tends to have higher C/N ratios than macrophytes, and thus tends to be of lower food quality. However, the increased structural tissues in woody plants may have rendered them more effective winter cache foods.

In extremely seasonal environments such as the High Arctic, herbivores must use plant resources in a highly efficient manner. Herbivores must consume the highest quality forage possible during the brief growing season to maximize nutrient and energy gain. High quality forage typically includes young leaves with high nitrogen content, minimal structural (fibrous) tissues, and low defense compound content^59,60^.

Within the Beaver Pond macrofossil assemblage, pod grass (an emergent macrophyte) and birch have the highest nitrogen content and lowest C/N (Fig. 5). A larger sample set is necessary to confirm this observation; however, current data supports the conclusion that emergent macrophytes and deciduous broadleaf trees were among the more nutritious types of forage available to *Dipoides* sp. at the Beaver Pond site. It should be noted that forage quality is not the only factor that governs herbivore feeding behaviour. Animals may preferentially target plants with higher biomass to minimize energy expenditure traveling between forage sites or select plants that grow in locations that minimize the risk of predation.

The C/N of high Arctic shrubs decreases over the course of the growing season^58^. As there is no time-constraint on macrofossil deposition at the Beaver Pond site, variation in C/N may also be due to differences in plant phenological stage at time of incorporation into the peat layer. The incorporation of senescent plants into the peat deposit at the end of each growing season may in part account for the lower than expected macrofossil N (wt%) values reported from this site.

### Beaver Pond site flora δ^13^C and δ^15^N

The Beaver Pond macrofossil assemblage contains a diverse range of terrestrial and freshwater plant species. The identified plant species in this study concur with previous interpretations that this was an open-forest landscape interspersed with shallow wetlands. Larch trees and cool-climate woody shrubs dominated the forest community. The wetlands supported both vascular macrophytes and dense assemblages of bryophytes.

The macrofossil *δ*^13^C are all within the range expected for primary producers utilizing the C_3_ photosynthetic pathway and accessing either ambient or dissolved atmospheric CO_2_ as their dominant carbon source. The *δ*^15^N of the macrofossils are also within the expected range for a riparian ecosystem in a cool climate biome.

While *Dipoides* sp. most likely consumed leafy tree branches and woody tissues (cambium), it is worth noting that plant seeds and cone bracts were analyzed in this study due to ease of macrofossil taxonomic identification. Leaf *δ*^13^C is typically lower than that of other plant parts^61^, although there is no clear pattern in intra-plant variation of *δ*^15^N.

### Moss

Samples of the dominant Beaver Pond site bryophyte, *Scorpidium* (hooked scorpion moss), have very low *δ*^13^C for a primary producer (− 36.6 to − 34.6‰). This pattern is consistent with modern mosses collected from freshwater habitats in Subarctic and Arctic regions^13,62–64^.

Environmental conditions dictate moss *δ*^13^C rather than species-specific physiological differences. Peat mosses can grow partially or fully submerged in water. Given that mid-Pliocene atmospheric CO_2_ concentration levels were similar to modern (~ 400 ppm)^15,65,66^, moss exposed to the atmosphere would preferentially have used the abundant ^12^CO_2_, resulting in low *δ*^13^C. Alternatively, low *δ*^13^C in peat mosses can also indicate an underwater growing environment rich in ^13^C-depleted respired CO_2_ from surrounding plants^62^.

Moss macrofossil *δ*^15^N is relatively high for a photosynthetic organism (mean =  + 4.8‰). This is indicative of either the presence of ^15^N-enriched sources of bioavailable N (i.e. dissolved nitrates, organic proteins such as urea or amino acids), or increased nutrient availability^45,61^. Unlike vascular plants, mosses do not uptake compounds through their roots. Rather, they obtain nutrients from wet or dry deposition through their leaves^67,68^. Today, beaver ponds are considered to be N sinks, with elevated rates of bacterially mediated denitrification^69^. These bacterial processes result in ^15^N-enriched products that are readily dissolved and used by plants (including moss) living in an aqueous environment. Decomposition processes also increase plant *δ*^15^N over time^47^ and remineralized organic debris decomposing in wetlands may be particularly ^15^N-enriched.

Beaver Pond bulk peat samples and moss macrofossils show a similar isotopic pattern (low *δ*^13^C and high *δ*^15^N), which suggests that hooked scorpion moss contributed substantially to peat biomass accumulation at the Beaver Pond site.

### Macrophytes

Beaver Pond macrophyte *δ*^13^C fall well within the albeit very wide known range for modern freshwater plants (− 50 to − 11‰, see Osmond et al.^70^, Keeley and Sandquist^54^, Mendonça et al.^55^, and Chappuis et al.^56^). It is reasonable to assume, however, that the very small sample size in this study hides the potential extent of the carbon isotope variability of macrophytes at the site.

Pod grass and bogbean are classified as emergent macrophytes (they grow rooted in water-logged substrates, but their leaves are exposed to the atmosphere), while pondweed grows entirely submerged. Submerged macrophytes become more enriched in ^13^C as the dissolved CO_2_ pool (the dominant carbon source) becomes increasingly limited^54^.

The Beaver Pond site pondweed *δ*^13^C is relatively low (− 26.5‰) for a submerged macrophyte. This indicates that it grew in an aquatic environment with adequate dissolved CO_2_. This is in keeping with the interpretation that the Beaver Pond was a fen (near neutral pH, cool water temperature) during the Pliocene. A low *δ*^13^C may also indicate high influx of terrestrial organic biomass or mosses (with low *δ*^13^C) into the water that subsequently remineralized and contributed to the dissolved inorganic carbon pool.

Environmental conditions strongly influence aquatic plant *δ*^15^N. Beaver Pond macrophyte *δ*^15^N (range =  + 0.2 to + 2.7‰) indicate interspecific access and use of a variety of different sources of bioavailable N within the water column and substrate. The most likely N sources are microbial-fixed atmospheric N_2_ (which ranges from –2 to + 2‰), the products of nitrification/denitrification processes (^15^N-enriched NH_4_^+^ or NO_x_), and remineralized ^15^N-enriched organic material (either terrestrial or aquatic)^71–73^.

### Larch

Larch (the extinct species *Larix groenlandii*) is the most common vascular plant species in this macrofossil assemblage.

There is an offset of ~ 2‰ between the *δ*^13^C of (i) larch shoots/buds (which bear the needles) and cone bracts, and (ii) larch seeds. Larch shoots and cones (*δ*^13^C range = ‒25.4 to − 25.1‰; mean = − 25.3‰) are more depleted of ^13^C than larch seeds (*δ*^13^C range = − 23.3 to − 22.7‰, mean = − 23.1‰). This could be indicative of seasonal physiological or environmental conditions experienced by larch trees at the Beaver Pond site. The cones and shoots of extant larch trees begin growing in the early spring and have lower *δ*^13^C, whereas their seeds (higher *δ*^13^C) do not develop and mature until mid to late summer^74^.

A number of physiological and environmental conditions could be responsible for this offset between needle/-bearing structures and seeds. Atmospheric vapor pressure deficit (aridity) induces stomatal closure in vascular plants^75^. This restricts not only the rate of water leaving the needle/tree, but also that of atmospheric CO_2_ entering it. Stomatal closure reduces CO_2_ entry and results in less discrimination against ^13^CO_2_. High levels of solar irradiance in the summer increase the rate of CO_2_ assimilation. Plants growing at very high latitudes experience 24-h of daylight during the summer. This creates a greater demand for CO_2_ to maintain photosynthesis and less discrimination against ^13^CO_2_. Both aridity and increased light levels could contribute to why Beaver Pond larch tissues grown late in the summer are more ^13^C-enriched than those grown in the early spring/the previous fall.

Alternatively, trees can use water and carbon (in the form of sugars) stored during the previous year to promote new growth during the early spring when leaves are absent and light levels are low. Tissues that develop early in the growing season (i.e. needle-bearing buds and shoots) can therefore reflect the *δ*^13^C of photosynthetic conditions from the previous growing season^76,77^. In addition, differences in the macromolecular (lipid, protein, sugar) composition of larch buds/needles versus seeds could account for their offset in *δ*^13^C (i.e. lipids are typically more ^13^C-depleted than proteins).

Larch *δ*^15^N (mean =  + 2.7‰) indicate that these conifer trees had access to N sources other than “light” fixed atmospheric N_2_. Given the proximity of wetlands, the root systems of larch trees may have had access to ^15^N-enriched dissolved nitrates in the surrounding water-logged soils. Increasing foliar N concentration due to atmospheric N deposition also drives up plant *δ*^15^N^78,79^.

Aridity may also have influenced terrestrial plants growing at the Beaver Pond site. Higher rainfall is inversely correlated with *δ*^15^N, where rainier ecosystems tend to produce more ^15^N-depleted plants^80^.

Similar to *δ*^13^C, there is an offset in *δ*^15^N (and N wt%) between larch needle-bearing structures (mean =  + 3.8‰; 0.9%) and larch seeds (mean =  + 2.1‰; 0.3%). This could indicate differences in the macromolecular composition of these different tissue types (where high N content typically indicates higher tissue protein content).

### Comparison of *Dipoides* within Castoridae

The composition of *Dipoides* sp. diet differs from that of other members of Castoridae that lived in North America during the late Cenozoic. Pliocene High Arctic *Dipoides* sp. (n = 5), modern subarctic *Castor canadensis* (n = 4) (Table 1), and late Pleistocene *Castoroides ohioensis* (n = 11) (Table 1) *δ*^13^C_col_ and *δ*^15^N_col_ are compared in Fig. 3. A correction for the Suess effect was first necessary render the *δ*^13^C of all three genera comparable. The carbon isotope composition of atmospheric CO_2_ has changed over time with global climatic conditions. More recently, anthropogenic burning of fossil fuels that has rapidly released CO_2_ enriched in ^12^C into the atmosphere^46,81^. Hence, a correction is needed when comparing *δ*^13^C of organic samples from different time periods to account for this isotopic variation in the primary carbon source of photosynthetic organisms at the base of the food web.

Suess effect corrections of + 2.02‰ and − 0.1‰ were applied to the *δ*^13^C_col_ of modern *C. canadensis* (collected in 2013 and 2014) and *Castoroides* (late Pleistocene in age), respectively. These corrections were based on the average *δ*^13^C of atmospheric CO_2_ (*δ*^13^C_CO2_) calculated from Pliocene dual-benthic and planktonic foraminifera proxy records, spanning from ~ 4.1 to 3.8 Ma (average *δ*^13^C_CO2_ = − 6.55‰)^76^. These foraminifera proxy records are approximately contemporary with the Beaver Pond site. Average *δ*^13^C_CO2_ for 2014 (− 8.57‰) was compiled from the Scripps CO_2_ monitoring program. Average *δ*^13^C_CO2_ for the late Pleistocene (− 6.45‰) was compiled using ice core data from Schmitt et al.^82^.

Plants growing during these three different time periods (Pliocene, late Pleistocene, and modern/2014) would reflect the *δ*^13^C of contemporary atmospheric CO_2_. Therefore, changes in *δ*^13^C_CO2_ help explain differences in *δ*^13^C between *Dipoides* sp. and modern *C. canadensis*. Additional factors, however, are important in explaining the wide range of *δ*^13^C and large enrichment in ^13^C measured for *Castoroides*.

*Dipoides* sp. diet composition differs from that of *Castoroides* (the Pleistocene giant beaver) (Figs. 3 and 7). *Castoroides*’ high *δ*^13^C_col_ and *δ*^15^N_col_ (mean *δ*^13^C_col_ = − 17.6‰ and mean *δ*^15^N_col_ =  + 5.8‰) indicate a diet composed predominantly of aquatic (particularly submerged) macrophytes and minimal woody plant material (Table 1)^13^.

In comparison with *Castoroides*, both *Dipoides* sp. and *C. canadensis* have a relatively small range of *δ*^13^C_col_ and *δ*^15^N_col_ (Table 1) (Fig. 3). *Dipoides* sp. mean *δ*^13^C_col_ and *δ*^15^N_col_ are higher than those of modern *C. canadensis* (Fig. 3). This is attributable to either variation in diet between the two species, or changes in global C and N baselines over geologic time.

Previous mixing model studies predict that extant *C. canadensis* diet is composed of approximately equal proportions of woody terrestrial plants and aquatic macrophytes^13^. However, this can vary by latitude and season. For example, extant *C. canadensis* in the Canadian subarctic vary their winter diet significantly depending on habitat^83^. It is worth noting that extant *C. canadensis* does not occur north of 70° latitude and High Arctic *Dipoides* sp. living at 78° latitude may have employed different dietary strategies.

*Dipoides* sp. may have relied more heavily than *C. canadensis* on underwater stores of tree branches to survive the long, dark polar winter. Tree bark is more ^13^C-enriched than leafy vegetation^57^ and increased consumption could account for the higher *δ*^13^C_col_ seen in *Dipoides* sp. Variation in the quantity and type of macrophytes consumed by each beaver species could also account for this difference (i.e. emergent and floating macrophytes are, on average more ^15^N-enriched than submerged macrophytes).

Changes in the isotopic composition of the C and N baseline between the Pliocene and the present could also account for the isotopic offset between beaver species. Further investigation of possible changes in the *δ*^15^N baseline of flora in terrestrial high latitude environments during the Pliocene would be a valuable avenue of future research.

### *Dipoides* sp. behaviour and evolutionary implications

**Figure 8:**
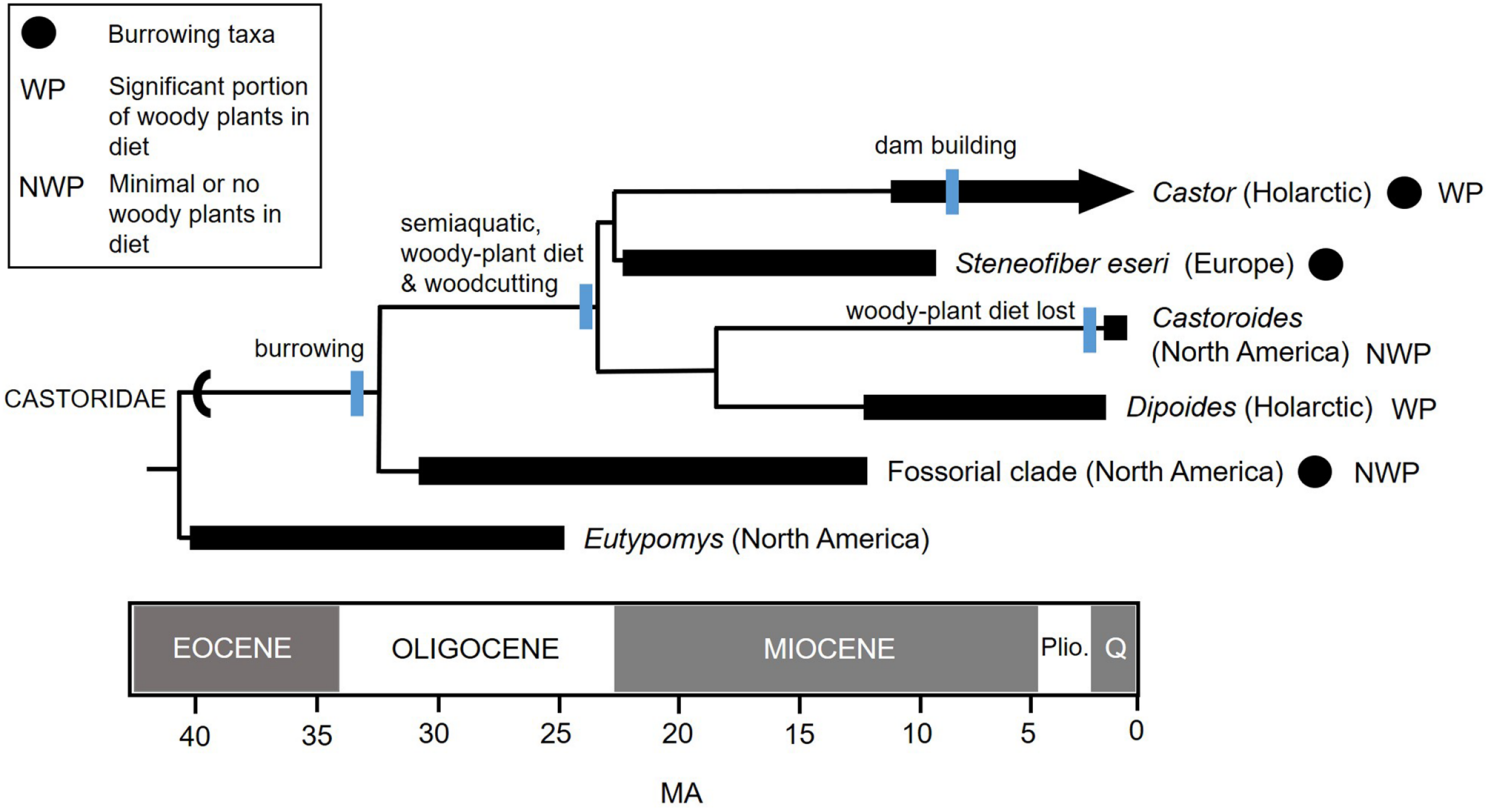
Simplified Castoridae phylogeny showing behavioural reconstructions, including new evidence of woody plant consumption in *Dipoides* sp. Diagram based on phylogenetic analysis by Rybczynski^9^, which used a matrix of 88 morphological characters and 38 taxa. The origination of dam building is a minimum age (~ 7–8 Ma), corresponding to the time of divergence of *Castor canadensis* and *C. fiber,* inferred from molecular evidence^96^ and supported by fossil evidence^97^. Legend: CIRCLE—taxa that burrowed (*Dipoides* and *Castoroides* may have burrowed, but direct fossil evidence is currently lacking); WP—taxa with significant woody plant contribution to their diet; NWP—taxa that did not generally consume woody plants (the terrestrial burrowing clade is associated with open plains and unforested habitat, and therefore assumed to have not consumed significant amounts of woody plants); Plio—Pliocene; Q—Quaternary. Age range sources: *Castor*^96,97,103^, nowdatabase.org; *Steneofiber eseri*^104^; Fossorial clade^84,86,94,105^; *Eutypomys*^94^, Fossilworks.org, nowdatabase.org; *Dipoides*, including *D. tanneri*: Fossilworks.org, nowdatabase.org; *Castoroides*^106^, Fossilworks.org, nowdatabase.org. Fossil taxa behavioural evidence sources: *Steneofiber eseri*^104^; *Castoroides*^13^; *Dipoides* (this study); Fossorial clade^90^.

Evidence from the Beaver Pond site has implications for our understanding of *Dipoides* sp. ecology. These data also contribute to our understanding of the evolution of behavioural transitions within Castoridae. In particular, how *Castor*’s distinctive complex of behavioural traits (tree harvesting, underwater food caching, and construction behaviour) may have evolved. A new hypothesis of behavioural evolution in castorids based on evidence from the fossil record (i.e. fossil burrows, cut wood, and stable isotope measurements) and skeletal-dental morphology is mapped onto a simplified phylogenetic tree in Fig. 8^42,84–87^.

Castoridae is a group of herbivorous rodents comprising roughly two dozen genera. Most fossil castorids fall within two major groups: a clade of fossorial specialists (Palaeocastorinae) and a semiaquatic clade^42,84,86,88–90^. The latter includes *Castor* and *Dipoides*. Members of the fossorial clade (~ 7 genera) possess striking specializations such large digging claws, extremely reduced tails, and broad, procumbent incisors for digging. In some cases, specimens have been found within fossil burrows (i.e. *Palaeocastor*, or “The devil’s corkscrew” burrows discovered in the plains of North America^88^). The semiaquatic group comprises two subfamilies, Castorinae (~ 6 genera, including *Steneofiber* and the extant *Castor*), and Castoroidinae (~ 7 genera, including *Dipoides* and the giant beaver, *Castoroides*). The oldest definitive Castorinae in the fossil record is *Steneofiber eseri* from the early Miocene (France, MN2, ~ 23 Ma). *S. eseri* shows evidence of living in family groups and swimming specializations^91^. This, in combination with aDNA evidence^12^, suggests Castorinae and Castoroidinae are derived from a semiaquatic ancestor in the early Miocene.

Digging behaviour was not just characteristic of the fossorial group and appears within the semiaquatic clade as well. *Castor*, though not morphologically highly specialized for the task, digs bank burrows and creates extensive canal systems^92^. In addition, the extinct semiaquatic beaver *Steneofiber eseri* was found within a burrow^91^. Considering the phylogenetic distribution of burrowing behaviour within the Castorid tree (Fig. 8), it is likely that the common ancestor of the fossorial and semiaquatic clades also burrowed. Thus, the appearance of burrowing behaviour within *Castor* and *Steneofiber* are seen as a retention of a primitive trait^9^.

If burrowing behaviour in semiaquatic castorids is the primitive condition, it is likely *Dipoides* burrowed as well, as seen in other semiaquatic rodents today such as *Castor*, but also *Crossomys* (earless water rat), *Myocastor* (nutria), and *Ondatra* (muskrat)^92^. It is also possible that *Dipoides* constructed lodges. Extant *Castor* and *Ondatra* are known to construct burrows and lodges, depending on the characteristics of the habitat. Bank burrows are associated with stream environments, whereas lodges are better suited to calmer waters^92^. Unlike *Castor,* extant *Ondatra* construct their push-up lodges using cattails and other fibrous vegetation rather than wood. The abundance of cut wood at the Beaver Pond site^11^ suggests that *Dipoides* sp. had the option to incorporate wood into their nesting structures, and possibly built lodges.

Given the occurrence of woodcutting and woody plant consumption within both subfamilies of semiaquatic castorids (represented by *Castor* and *Dipoides* in Fig. 8), it seems likely these behaviours appeared in the common ancestor of the semiaquatic group. Woody plant consumption may have preadapted castorids to exploit colder environments that arose during and after the late Miocene. *Castor canadensis* does not hibernate, but builds and sink rafts of branches and foliage to use as a source of fresh food during the winter months^1,93^. *Dipoides* sp. may have also engaged in this behaviour and used underwater caches of branches as a primary food source to survive the consecutive months of darkness during the high latitude winter when plants become dormant. The use of woody plants in this way may have been key to allowing beavers to disperse between North American and Eurasia, which required crossing the Bering Isthmus^94^, a high latitude landmass. Curiously, given that a diet rich in woody plants appears to be the primitive condition of semiaquatic castorids, the absence of woody plant consumption seen in the Pleistocene giant beaver *Castoroides*^13^ must be interpreted here as an evolutionary loss and potentially a leading factor in their extinction (Fig. 8).

Among living mammals, *Castor*’s dam construction is a unique and highly derived behaviour – an evolutionary puzzle, associated with a set of innate behavioural specializations^95^. For example, dam construction is well known to be triggered by the sound of running water alone^95^. The presence of such “hard-wired” behaviours may be associated with the ancient origins of this behaviour. Molecular and fossil occurrence records indicate that the split between Eurasian and North American *Castor* arose around 7.5 Ma ago^96,97^, implying that dam building behaviour itself is at least as old.

Definitive fossil evidence for dam building by an extinct beaver is currently lacking. Consequently, dam building behaviour is shown as possibly arising only on the lineage leading to *Castor*. Hypothetically, dam building may have arisen from beavers collecting branches near their burrow/lodge for feeding purposes and the accumulations of sticks could have dammed streams by happenstance. The effects may have been multifold. A deeper pond is an effective defense mechanism and provides a safe refuge from predators. Raised water levels also create more favourable conditions for underwater food caching of branches in sub-freezing winter conditions because the deeper water would prevent an underwater food cache from being locked in ice. As such, natural selection would have favoured animals that maintained the dam, presumably as an extension of their pre-existing nesting behaviour such as lodge building. In this scenario, the climate cooling that started around 15 Ma ago and continued into the Pleistocene would have provided an interval where behaviours promoting over-wintering survival, such as underwater food caching branches and dam building, would have been increasingly reinforced by natural selection.

It seems unlikely that the common ancestor of all semiaquatic beavers was a dam-builder. Extant *Castor* is a large powerful rodent weighing 12–25 kg, with some individuals as large as 40 kg^92^. Its body size is one factor that allows the animal to harvest branches and whole trees to build lodges and maintain dams over multiple years. The Beaver Pond site *Dipoides* sp. was also a large rodent and was roughly two-thirds the size of an average extant *Castor*. In contrast, the less-derived semiaquatic beavers, such as the Miocene *Eucastor tortus* (Castoroidinae) and *Steneofiber eseri* (Castorinae) were small (~ 1 kg, or less), suggesting that the common ancestor of the semiaquatic lineage was also small bodied. Although the common ancestor of the semiaquatic beaver lineage is inferred to have consumed woody plants (this study), and may have used branches in creating food piles and wood for lodge construction, it would have been too small to have had the capacity to build and maintain dams. As such, if *Dipoides* sp. did exhibit dam building behaviour, it would be the result of parallel evolution within the Castoroidinae and Castorinae lineages.

## Conclusions

Here, we reconstruct Pliocene High Arctic *Dipoides* sp. palaeodiet from bone collagen *δ*^13^C and *δ*^15^N within the context of an isotopic dietary baseline composed of coeval ~ 4 Ma old terrestrial and freshwater plant macrofossil remains. The Beaver Pond site provides a very rare opportunity for such a palaeodiet reconstruction using coeval herbivore and plant remains. A Bayesian mixing model indicates that *Dipoides* sp. diet was composed of approximately equal proportions of woody plant material and freshwater macrophytes, with slightly more emphasis on macrophyte consumption. *Dipoides* sp. dietary preferences lie somewhere in between those of other North American late Cenozoic semiaquatic beavers (extant *Castor* and extinct Pleistocene giant beaver, *Castoroides*).

The consumption of woody plant material suggests that a proportion of the assemblage of the wood cut by *Dipoides* sp. at the Beaver Pond fossil site was the result of harvesting for consumption, possibly as part of an underwater winter food cache. The results also suggest that the early Miocene ancestor of the semiaquatic beaver lineage engaged in woodcutting and consumed woody plants as part of its diet. Swimming, woodcutting, and a diet of woody plants could have set the stage for the evolution of dam building behaviours—advantageous traits that may have been selected for by the cooling climate of the late Neogene, and which have resulted in *Castor’s* modern role as a keystone species and ecosystem engineer.

## Methods

The *Dipoides* sp. skeletal material and the plant macrofossils used in this study originated from the Beaver Pond fossil site, Unit III, as defined by Mitchell et al.^21^. Unit III is a peat layer that yielded the majority of the beaver-cut sticks and vertebrate faunal remains discovered at the site. It is interpreted to have been a rich fen connected to open water, within a larch-dominated forest ecosystem^21^.

### Plant macrofossil preparation

Plant macrofossils were isolated from bulk samples of Unit III peat and identified to taxon. Macrofossils were extracted from the peat using a combination of water-flotation and wet-sieving. Organic material greater than 0.425 mm was retained for further cleaning. Adhered sediment and moss were removed from the macrofossils using surgical forceps and repeated ultrasonic water baths. Cleaned macrofossils were dried at 26 °C for 24 h and identified to taxon using a binocular microscope.

### Stable isotope analysis

*Dipoides* sp. bone collagen was extracted and its *δ*^13^C and *δ*^15^N measured at the Alaska Stable Isotope Facility (UAF). Collagen extraction was performed using a modified Longin^98^ method of gelatinization. Organic contaminants were removed using XAD-2 resin^99,100^ and collagen purification was performed according to methods developed by Matheus^101^. Non-soluble collagenous portions were rinsed to neutral pH, but not subjected to base treatment. Collagen was gelatinized in weak HCl (pH 3) under N_2_ gas at 105° C until dissolved (2 to 6 h). The solution was centrifuged and filtered with a 0.45 µm syringe-type PTFE filter and the supernatant containing dissolved collagen was lyophilized and weighed to determine the percent collagen yield. Lyophilized collagen was then hydrolyzed in 6 N HCL under N_2_ gas for 4 h at 120 °C. The hydrolyzates were passed by gravity flow through 2 cc of compacted Serva XAD-2 HPLC resin in syringe columns to extract humates and other long-chain organic contaminants that can adhere to fossil collagen. The hydrolyzates were passed through a 0.45 µm PTFE filter placed at the distal end of each syringe column and were dried by rotary evaporation. Stable carbon and nitrogen isotope analysis of the hydrolyzed collagen was performed using a GC-Isolink gas chromatography combustion system coupled to a Thermo Scientific Delta V Plus isotope ratio mass spectrometer operated in continuous flow mode, using helium as the carrier gas.

Plant macrofossils were powdered using a ball-bearing mill and weighed into tin capsules (0.38 ± 0.02 mg). Stable carbon and nitrogen isotope analysis of macrofossil remains was conducted at the LSIS-AFAR facility at the University of Western Ontario (London, Canada). Samples were analyzed in continuous flow mode using a Costech elemental analyzer (ECS 4010), coupled to a Thermo Scientific ConFlo IV and Delta V Plus isotope ratio mass spectrometer in continuous flow mode, using helium as the carrier gas. One method duplicate (complete duplication of sample preparation and isotopic analysis) and one analytical duplicate (separate isotopic analysis of sample powder) were included for every ten samples. The carbon and nitrogen isotope measurements of the plant macrofossils were completed in separate analytical sessions. The first session was used to determine *δ*^13^C and nitrogen content (weight percent, N wt%); values of *δ*^15^N were determined in the second session, using individually tailored weights based on each sample’s N wt%.

All isotopic results are reported in *δ*-notation in per mil (‰) relative to international standards. Collagen *δ*^13^C and *δ*^15^N were calibrated to VPDB and AIR, respectively. Analytical accuracy and precision were 0.0‰ for *δ*^13^C measurements, and 0.2‰ for *δ*^15^N measurements.

Plant macrofossil *δ*^13^C and *δ*^15^N were calibrated to VPDB and AIR, respectively using USGS40 (accepted *δ*^13^C = − 26.39‰, SD =  ± 0.0‰; accepted *δ*^15^N = − 4.52‰, SD =  ± 0.2‰) and USGS41a (accepted *δ*^13^C =  + 36.55‰, SD =  ± 0.1‰; accepted *δ*^15^N =  + 47.55‰, SD =  ± 0.2‰). Additional reference materials IAEA-CH-6 (accepted *δ*^13^C = − 10.45‰, SD =  ± 0.0‰) and NIST-1547 (Peach Leaves) (internally calibrated *δ*^15^N =  + 1.98‰, SD =  ± 0.1‰) were used to evaluate instrument precision and accuracy for *δ*^13^C and *δ*^15^N, respectively. A keratin powder (Spectrum Chemicals Mfg. Corp., derived from pig skin and hair) was also included to monitor instrument drift. Combined instrument and analytical errors were ± 0.1‰ for *δ*^13^C, and ± 0.2‰ for *δ*^15^N.

The *Dipoides* sp. and plant macrofossil isotopic results were incorporated into a statistically-based Bayesian mixing model (SIAR V4). This approach provides a statistically robust means of evaluating the relative dietary contributions of woody plants and aquatic primary producers to *Dipoides* sp. Diet^102^.

## Data Availability

The authors declare no limitations on data or standard operating protocol availability.
